# Acute chest syndrome, airway inflammation and lung function in sickle cell disease

**DOI:** 10.1371/journal.pone.0283349

**Published:** 2023-03-30

**Authors:** Aliva De, Sanford Williams, Yujing Yao, Zhezhen Jin, Gary M. Brittenham, Meyer Kattan, Stephanie Lovinsky-Desir, Margaret T. Lee

**Affiliations:** 1 Division of Pediatric Pulmonology, Vagelos College of Physicians and Surgeons, Columbia University Irving Medical Center, New York, NY, United States of America; 2 Department of Pediatrics, Columbia University Irving Medical Center, New York, NY, United States of America; 3 Department of Biostatistics, Mailman School of Public Health, Columbia University, New York, NY, United States of America; 4 Division of Pediatric Hematology/Oncology and Stem Cell Transplantation, Vagelos College of Physicians and Surgeons, Columbia University Irving Medical Center, New York, NY, United States of America; University of Illinois at Chicago, UNITED STATES

## Abstract

**Background:**

Acute chest syndrome (ACS) is an acute complication in SCD but its effects on lung function are not well understood. Inflammation is a key component of SCD pathophysiology but with an unclear association with lung function. We hypothesized that children with ACS had worse lung function than children without ACS and aimed to investigate the association of lung function deficits with inflammatory cytokines.

**Methods:**

Patients enrolled in a previous 2-year randomized clinical trial who had consented to future data use, were enrolled for the present exploratory study. Patients were categorized into ACS and non-ACS groups. Demographic and clinical information were collected. Serum samples were used for quantification of serum cytokines and leukotriene B4 levels and pulmonary function tests (PFTs) were assessed.

**Results:**

Children with ACS had lower total lung capacity (TLC) at baseline and at 2 years, with a significant decline in forced expiratory volume in 1 sec (FEV1) and mid-maximal expiratory flow rate (FEF25-75%) in the 2 year period (p = 0.015 and p = 0.039 respectively). For children with ACS, serum cytokines IL-5, and IL-13 were higher at baseline and at 2 years compared to children with no ACS. IP-10 and IL-6 were negatively correlated with PFT markers. In multivariable regression using generalized estimating equation approach for factors predicting lung function, age was significantly associated FEV1 (p = 0.047) and ratio of FEV1 and forced vital capacity (FVC)- FEV1/FVC ratio (p = 0.006); males had lower FEV1/FVC (p = 0.035) and higher TLC (p = 0.031). Asthma status was associated with FEV1 (p = 0.017) and FVC (p = 0.022); history of ACS was significantly associated with TLC (p = 0.027).

**Conclusion:**

Pulmonary function abnormalities were more common and inflammatory markers were elevated in patients with ACS, compared with those without ACS. These findings suggest airway inflammation is present in children with SCD and ACS, which could be contributing to impaired pulmonary function.

## Introduction

The pathophysiological processes in sickle cell disease (SCD) are multicellular and multi-factorial [1–6]. Pulmonary complications cause significant morbidity and mortality in children and adults with SCD [7–10]. Acute chest syndrome (ACS), an acute complication in SCD [11], is frequently precipitated by pulmonary infections and inflammation, and complicated by factors such as fat emboli to the lungs, pulmonary infarction, hypoventilation from pain and regional atelectasis [7]. A range of pulmonary function test (PFT) abnormalities have been described in SCD including obstructive defects, airway hyper-responsiveness, restrictive defects and diffusion defects [12–18]. The consequences of ACS on future lung function have not been well characterized, with contradictory reports of an association of ACS with a decline in lung function [19–23]. Asthma is a well-known comorbidity in SCD [24–28]. However, PFT deficits in SCD are seen irrespective of asthma status [29], with poorly understood underlying mechanisms.

The role of inflammatory mediators is increasingly recognized in the pathogenesis of SCD complications, in concert with HbS polymerization-vaso-occlusion and hemolysis-endothelial dysfunction [29, 30]. Some preliminary data from animal studies separately investigating effects of ischemia reperfusion injury and allergic sensitization on pulmonary inflammation, and a handful of human studies have elucidated pathways involving NKT cell activation, Th1 (TNFa, IFN-γ, IP-10, Th-17), Th2 (IL-4, IL-5, IL-13) and monocytic (IL-6 and monocyte chemotactic protein) inflammation [18, 29, 31]. Th2 inflammatory cytokines are classically associated with asthma and atopy. The inflammatory chemokine interferon-gamma inducible protein (IP-10), also known as C-X-C motif chemokine ligand 10 (CXCL10), is a biomarker associated with several inflammatory disorders and associated with respiratory infections [32]. In a previous study, we investigated inflammatory patterns in children with SCD and history of asthma and ACS, in comparison to non-SCD controls with classic allergic asthma and demonstrated elevated Th1 (TNFa, and IP-10), Th2 (IL-4) and monocytic markers in the SCD cohort and an inverse relationship between lung function and Th1 markers (IP-10 and TNFa) [18]. It is not known if airway inflammation and lung function deficits in SCD are secondary to underlying systemic inflammation or a result of pulmonary injury following ACS or allergic diseases.

With the present exploratory study, using PFT data and serum samples collected from a cohort of patients from a previous clinical trial of vitamin D therapy in children with SCD (ViDAS) [33], we hypothesized that a history of ACS in children with SCD is associated with pulmonary inflammation and lung function deficits. We further hypothesized that lung function worsens over time for patients with ACS and reviewed data from the 2-year follow up period in the ViDAS clinical trial after adjusting for the clinical trial intervention.

## Methods

### Study population

The study population consisted of a subset of patients from a 2-year clinical trial study at the Sickle Cell Clinic at Morgan Stanley Children’s Hospital, Columbia University Irving Medical Center (CUIMC), that investigated the effects of monthly high dose Vitamin D3 (100,000 IU/month- Arm A) and standard dose (12,000 IU/month- Arm B) to prevent respiratory complications, including respiratory infections, asthma exacerbations and ACS [33]. Both groups had reduced respiratory events at 2 years with no difference between groups. In the current exploratory study, we used serum samples and PFT data from 55 study participants (age 3–18 years, 26 males) who had consented to future studies. This study was approved by the Human Subjects Research Office and Institutional Review Board at CUIMC and consent was waived. We collected demographic and baseline clinical characteristics including age, sex, BMI, ethnicity, type of sickle cell disease, history of ACS, asthma, allergic rhinitis, eczema, family history of asthma, and use of hydroxyurea, from medical records review. Patients were categorized in ACS and non-ACS groups based on clinical information provided at baseline. None of the children in the non-ACS group developed ACS during the 2-year ViDAS clinical trial study period.

### Study procedures

#### Quantification of serum cytokines

Serum levels of Th1 inflammation (IFN-γ, TNFa, IP-10, Th-17), Th2 inflammation (IL-4, IL-5, IL-13), and monocyte activation (MCP-1, and IL-6) were measured using Milliplex human cytokine/Chemokine/Growth factor magnetic bead panel A (MilliporeSigma, St. Louis, MO) and the Luminex 200 platform (Luminex Corp, Austin, TX). The samples were processed according to the manufacturer’s instructions and the cytokine concentrations were quantitated by the Luminex xPONENT v3.1 and MILLIPLEX Analyst v5.

#### Pulmonary function tests

PFTs that were performed as part of the ViDAS trial included spirometry [forced expiratory volume in 1 second (FEV1), forced vital capacity (FVC), forced expiratory flow rate at 25–75% (FEF25-75%), ratio of FEV1 and FVC (FEV1/FVC)] lung volume measurements (total lung capacity (TLC), residual volume (RV)], diffusing capacity of the lungs for carbon monoxide (D_L_CO), and fractional exhaled nitric oxide (FeNO). PFTs were performed at baseline and annually for 2 years. Baseline and 2 year PFTs were used for the current analysis.

### Statistical analysis

The comparison of demographic, clinical characteristics and PFTs between the ACS group and the non-ACS group was performed using Wilcoxon rank sum test for continuous variables and Fisher’s exact test for categorical variables. All cytokine levels were log10 transformed. Wilcoxon signed rank test was used to compare cytokines and PFTs from baseline and 2-year follow up for ACS and non-ACS groups separately. Spearman’s rank correlation was used to investigate the correlation between cytokines levels and PFT parameters. Multivariable linear regression models were performed to evaluate the association between differences in PFT parameters and inflammatory markers between baseline and 2 years after adjustment for the intervention (Vitamin D supplementation). The generalized estimating equation (GEE) approach with identity link and working independence correlation structure was used to investigate the effect of ACS and other factors on lung function at baseline and 2 years. The covariates that were significant at the significance level of 0.05 in the univariable analysis (age and FEV1/FVC; asthma and FVC, FEV1; ACS and TLC; randomization and FVC) and additional known confounders were used in the multivariable GEE model. The covariates included age, sex, ACS, asthma, hydroxyurea, inflammatory patterns and lung function and treatment arms. Additional sensitivity analysis with SCD genotype as a covariate was done. Variables with p-value < 0.05 were considered significant. Analysis was performed using R 4.0 and SAS 9.4 (Cary, NC).

## Results

The baseline demographic characteristics of 55 children, randomized to two treatment arms in the ViDAS trial are shown in Table 1. At baseline, 40 patients (72.7%) had history of ACS, 15 (27.3%) had asthma, and 6 (10%) had allergic rhinitis. Thirty-eight (71.7%) children were on hydroxyurea. There were no differences in demographical and clinical characteristics between ACS and non-ACS groups.

**Table 1:** Demographic characteristics at baseline.

	ALL	Non-ACS group	ACS group	
	N = 55	N = 15 (27.3%)	N = 40 (72.7%)	p-value
Age at enrollment (years), mean ± SD	9.58 ± 3.85	9.47 ± 4.93	9.62 ± 3.44,	0.783
Male, n (%)	26 (47.3)	5 (33.3)	21 (52.5)	0.239
BMI*, median [min, max]	17.12 [11.65, 28.79]	18.60 [12.77, 28.79]	16.85 [11.65, 24.96]	0.320
Ethnicity, n (%) Hispanic	28 (50.9)	9 (60.0)	19 (47.5)	0.547
Type of sickle cell (%) HbSS HbSC HbSB Th HbS/HPFH	47 (85.5) 5 (9.1) 2 (3.6) 1 (1.8)	11 (73.3) 2 (13.3) 1 (6.7) 1 (6.7)	36 (90.0) 3 (7.5) 1 (2.5) 0 (0.0)	0.221
Eczema, n (%)	3 (5.5)	1 (6.7)	2 (5.0)	1.000
Allergic rhinitis, n (%)	6 (10.9)	2 (13.3)	4 (10)	0.660
Asthma, n (%)	16 (29.1)	3 (20.0)	13 (32.5)	0.510
Family history of asthma, n (%)	12 (21.8)	3 (20.0)	9 (22.5)	1.000
Hydroxyurea^Ɨ^	38 (71.7)	8 (61.5)	30 (75.0)	0.480
Vitamin D*	17.35 ± 8.72	17.93 ± 8.80	17.13 ± 8.79	0.779
Randomization, n (%) arm A—100,000 IU/month arm B—12,000 IU/month	27 (49.1) 28 (50.9)	6 (40.0) 9 (60.0)	21 (52.5) 19 (47.5)	0.547

* n = 54, Ɨ n = 53

BMI, body mass index; SD, standard deviation

For participants with PFT data (n = 54), 26.9% had obstructive defects (FEV1/FVC <80%) at baseline and 35.2% at 2 years (p = 0.012). A restrictive lung defect (TLC <80%) was present in 48.6% and 62.8% of participants at baseline and at 2 years (p = 0.049) respectively. Changes in PFT parameters from baseline to 2 years for the ACS and non-ACS groups were significant for a decline in FEV1 (p = 0.015) and FEF25-75% (p = 0.039) in the ACS group compared to the non-ACS group (Table 2). This significant decline in FEF25-75% (p = 0.007) was particularly noted in patients with multiple episodes of ACS (defined as more than one episode) compared to a single episode of ACS (Table 3). FEV1 and DLCO were also lower in patients with multiple ACS episodes at 2 years though not statistically significant (p = 0.079 and p = 0.090 respectively). For the ACS group, TLC was lower at baseline (ACS, 77.50 [61.00, 114.00] vs non-ACS 95.00 [69.00, 134.00], p = 0.034), and at 2 years (ACS, 76.00 [61.00, 130.00] vs non-ACS, 85.50 [69.00, 104.00], p = 0.055) (Supplemental Table 1a and 1b in S1 Table).

**Table 2:** Descriptive statistics of changes of PFT parameters from baseline to 2 years for ACS vs. non- ACS groups.

Parameters	n	Non-ACS Median [min, max]	ACS Median [min, max]	Wilcoxon rank sum test p-value
FVC % predicted	51	2.00 [-4.00, 16.00]	-1.00 [-21.00, 17.00]	0.078
FEV1% predicted	51	2.00 [-9.00, 18.00]	-3.00 [-23.00, 7.00]	**0.015**
FEV1/FVC	51	0.00 [-10.00, 5.00]	-2.50 [-24.00, 13.00]	0.386
FEF25-75% predicted	45	0.50 [-14.00, 38.00]	-10.00 [-109.00, 42.00]	**0.039**
TLC % predicted	33	0.00 [-51.00, 4.00]	-2.00 [-25.00, 12.00]	0.758
DLCO adj for Hb (ml/min^-1^mmHg^-1^)	28	4.50 [-26.00, 20.00]	1.50 [-41.00, 40.00]	0.780
KCO adj for Hb (ml/min^-1^mmHg^-1^L^-1^)	30	4.00 [-28.00, 24.00]	7.00 [-38.00, 29.00]	0.507

ACS, acute chest syndrome; FVC, forced vital capacity; FEV1, forced expiratory volume in 1 second; FEF25-75%, forced expiratory flow rate at 25–75% flow; TLC, total lung capacity; DLCO adj for Hb, diffusing capacity of the lung for carbon oxide adjusted for hemoglobin; KCO, carbon monoxide transfer coefficient

**Table 3:** Descriptive statistics of changes of PFT parameters from baseline to 2 years for single ACS and multiple episodes of ACS.

Parameters	n	Single ACS episode Median [min, max]	Multiple ACS episodes Median [min, max]	Wilcoxon rank sum test p-value
FVC % predicted	38	-1.00 [-21.00, 17.00]	-1.00 [-11.00, 15.00]	0.988
FEV1% predicted	38	0.00 [-12.00, 7.00]	-4.00 [-23.00, 6.00]	0.079
FEV1/FVC	38	0.00 [-14.00, 13.00]	-3.00 [-24.00, 5.00]	0.155
FEF25-75% predicted	35	-1.00 [-43.80, 42.00]	-13.50 [-109.00, 1.00]	**0.007**
TLC % predicted	26	1.50 [-24.00, 12.00]	-5.00 [-25.00, 1.00]	0.114
DLCO adj for Hb (ml/min^-1^mmHg^-1^)	20	7.50 [-41.00, 40.00]	-4.50 [-28.00, 13.00]	0.090
KCO adj for Hb (ml/min^-1^mmHg^-1^L^-1^)	23	6.50 [-38.00, 29.00]	9.00 [-6.00, 26.00]	0.549

ACS, acute chest syndrome; FVC, forced vital capacity; FEV1, forced expiratory volume in 1 second; FEF25-75%, forced expiratory flow rate at 25–75% flow; TLC, total lung capacity; DLCO adj for Hb, diffusing capacity of the lung for carbon oxide adjusted for hemoglobin; KCO, carbon monoxide transfer coefficient

**Fig 1:**
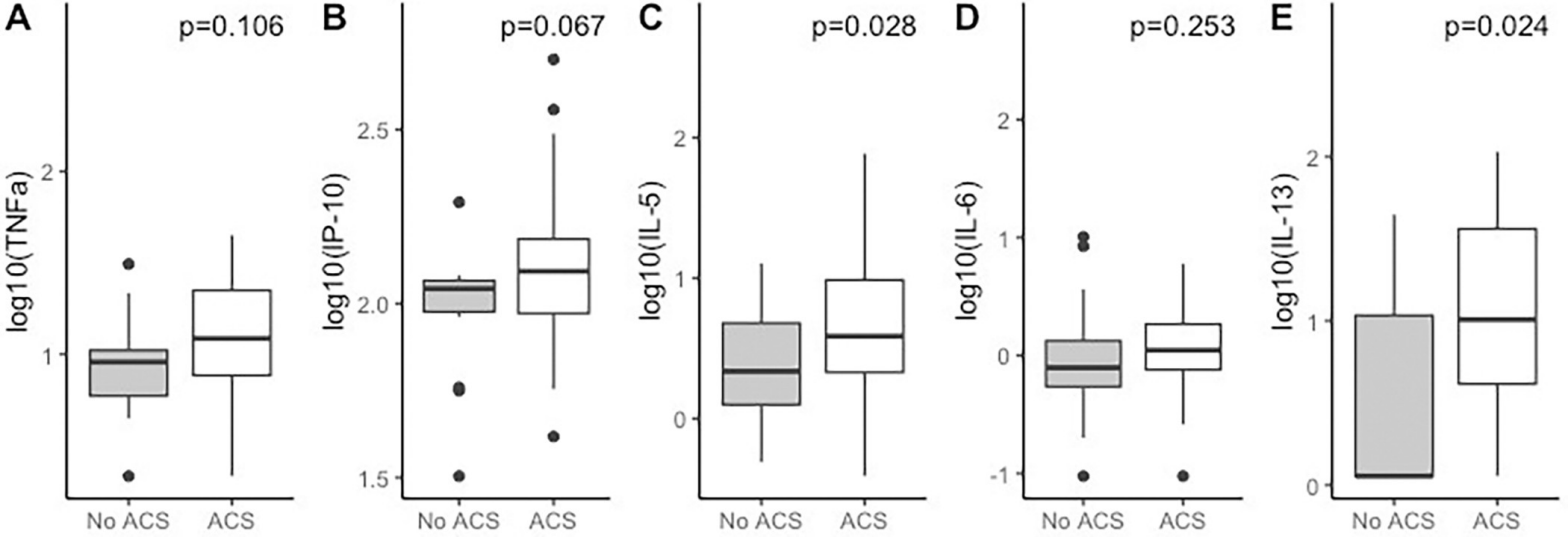
Cytokine levels at baseline for ACS vs non-ACS groups. Boxplots of TNFa, IP-10, IL-5, IL-6 and IL-13 at 2 years for two ACS groups (non-ACS: n = 15; ACS: n = 40) with p-value obtained from Wilcoxon rank sum test.

**Fig 2:**
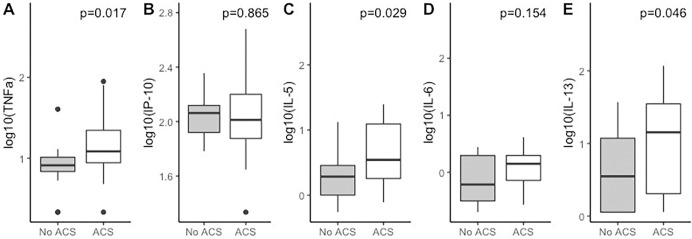
Cytokine levels at 2 years for ACS vs non-ACS groups. Boxplots of TNFa, IP-10, IL-5, IL-6 and IL-13 at 2 years for two ACS groups (non-ACS: n = 15; ACS: n = 40) with p-value obtained from Wilcoxon rank sum test.

At baseline, for children with ACS, IL5 (ACS, 0.59 [-0.41, 1.89] vs non-ACS, 0.34 [-0.31, 1.11], p = 0.028) and IL-13 (ACS 1.01 [0.05, 2.03] vs non-ACS: 0.05 [0.05, 1.65], p = 0.024) levels were higher (Fig 1). IP-10 was higher for children with ACS, but did not reach statistical significance (vs ACS 2.09 [1.62, 2.70] vs non-ACS: 2.04 [1.50, 2.29], p = 0.067). Fig 2 shows the difference in these cytokine levels for ACS vs non-ACS groups at 2 years, with elevated IL-5 (ACS, 0.54 [-0.11, 1.40] vs non-ACS, 0.29 [-0.26, 1.12], p = 0.029), IL-13 (ACS 1.15 [0.05, 2.07] vs no ACS: 0.55 [0.05, 1.57], p = 0.046) and TNF (ACS, 1.08 [0.33, 1.95] vs non-ACS: 0.91 [0.33, 1.61], p = 0.017) in patients with ACS. There were no significant changes in individual cytokine levels from baseline to 2 years for patients with ACS vs no ACS (S1 Fig).

Linear regression model for the association between changes of FVC, FEV1, and TLC with changes of inflammatory markers, using treatment arms as a covariate for the ACS group (Table 4) showed IP10 to be inversely associated with FVC (coefficient: -10.75, p = 0.05). IL-6 was inversely associated with FVC and positively associated with FEV1/FVC but did not reach statistical significance, (coefficient: -5.97 and 5.46, p = 0.06).

**Table 4:** Relationship of cytokines and PFT parameters for ACS group.

Log10 transformed Cytokines(pg/ml)/	FVC Estimate(p-value)	FEV1 Estimate(p-value)	FEV1/ FVC Estimate(p-value)	TLC Estimate(p-value)
TNFa	0.92 (0.85)	0.34 (0.93)	0.61 (0.89)	3.43 (0.67)
IL-5	0.50 (0.90)	2.18 (0.45)	1.12 (0.75)	-1.49 (0.85)
IL-6	-5.97 (0.06)	-0.34 (0.89)	5.46 (0.06)	3.16 (0.57)
IP-10	-10.75 **(0.05)**	-4.83 (0.26)	6.43 (0.20)	7.09 (0.41)
IL-13	-1.94 (0.51)	-0.69 (0.76)	2.47 (0.35)	2.27 (0.63)

Linear regression model for the association between changes of FVC, FEV1, FVC, TLC and changes of inflammatory markers with treatment as covariates for the ACS group.

e.g., change of FVC = b0 + b1*trt + b2*change of log10(TNFa) + error. The table shows the estimates of b2 for different PFT parameter outcomes and different biomarkers.

To investigate the association of ACS and lung function, multivariable GEE modeling with natural link and working independence correlation structure was used along with investigation of other confounding predictors, including, age, sex, asthma, hydroxyurea, and treatment arms. Age was significantly associated FEV1 (-0.881, 95%CI: (-1.75, -0.014), p = 0.047) and FEV1/FVC (-0.611, 95%CI: (-1.043, -0.179), p = 0.006). Compared to females, males had significantly lower FEV1/FVC (-3.42, 95%CI: (-6.60, -0.24), p = 0.035) and higher TLC (8.54, 95%CI: (0.758, 16.3), p = 0.031). Asthma status was significantly associated with FEV1 (-8.98, 95%CI: (-16.38, -1.58), p = 0.017) and FVC (-8.850, 95%CI: (-16.4, -1.25), p = 0.022); history of ACS was significantly associated with TLC (-10.9, 95%CI: (-20.5, -1.24), p = 0.027); and treatment arm was significantly associated with FVC (-6.879, 95%CI: (-13.49, -0.272), p = 0.041) (Table 5). Additional sensitivity analysis with genotype as a covariate demonstrated that the magnitude of the association between history of ACS and TLC was slightly diminished likely due to reduced power (S2 Table). Age became a predictor for TLC.

**Table 5:** Multivariable GEE model for predictors of lung function parameters.

Outcome	FEV1		FVC		FEV1/FVC		TLC	
Variable	Coefficient (95%CI)	p-value	Coefficient (95%CI)	p-value	Coefficient (95%CI)	p-value	Coefficient (95%CI)	p-value
Age at enrollment (years)	-0.881 (-1.75, -0.014)	**0.047**	-0.331(-1.17, 0.506)	0.438	-0.611(-1.043, -0.179)	**0.006**	1.032(-0.237, 2.30)	0.111
Sex Male Female	0.629(-5.40, 6.65) Reference	0.838	1.75(-4.62,8.12) Reference	0.590	-3.42(-6.60, -0.24) Reference	**0.035**	8.54(0.758, 16.3) Reference	**0.031**
Asthma Y N	-8.98(-16.38, -1.58) Reference	**0.017**	-8.850(-16.4, -1.25) Reference	**0.022**	-0.311(-3.95, 3.33) Reference	0.867	-3.01(-11.5,5.52) Reference	0.489
H/o ACS Y N	-0.830(-7.908, 6.248) Reference	0.818	-0.607(-8.56, 7.34) Reference	0.881	-0.464(-4.52, 3.60) Reference	0.823	-10.9(-20.5, -1.24) Reference	**0.027**
Hydroxyurea Y N	0.330(-6.42, 7.08) Reference	0.924	1.01(-6.28, 8.30) Reference	0.786	-0.278 (-4.15, 3.59) Reference	0.888	0.465 (-9.23, 9.35) Reference	0.992
Randomization A B	-5.55(-11.88, 0.772) Reference	0.085	-6.879 (-13.49, -0.272) Reference	**0.041**	1.18(-1.98, 4.34) Reference	0.465	2.46(-10.6, 5.67) Reference	0.555

GEE model with identity link and independence correlation structure to predict PFT parameters using age, sex, ACS, asthma, hydroxyurea and treatment arms

## Discussion

We compared lung function parameters and inflammatory markers in children with SCD with and without ACS.TLC was significantly lower for children with ACS at baseline and at 2 year follow up. Patients with ACS also showed changes in flow rates over a 2-year period, which was particularly seen in children with multiple episodes of ACS. Elevated concentrations of IL-5 and IL-13, which persisted at 2 years along with elevated TNFa at 2 years, suggested that Th1 and Th2 markers are elevated with ACS. There was a trend towards elevated IP10 for children with ACS at baseline. Changes in IP10 negatively correlated with changes in FVC for patients with ACS. Multivariable modeling found ACS to be a predictor of TLC. Taken together these findings suggest that inflammatory markers are elevated in patients with ACS, and ACS was a predictor for low lung function, particularly TLC.

A decline in lung function over time in children with SCD has been previously reported in several studies [12, 13, 22, 34]. In the study by Cohen et al., previous episodes of ACS did not predict future pulmonary function or morbidity [20]. Patients with recurrent ACS had lower lung function parameters than patients without ACS in the study by Knight-Madden et al. [21], which align with our findings of lung function decline with multiple episodes of ACS. Our study patients also showed a decrease in FVC over 2 years in the ACS group but this was not statistically significant (p = 0.078). A similar pattern of decreased FVC but not TLC was also seen in a small group of patients in the study by Cohen et al. [20]. Cohen et al. suggest that this could be secondary to airway disease, scattered closing of diseased airways at variable rates and volume de-recruitment. For our study, we can also speculate that changes in TLC may take longer, and a 2-year interval might be insufficient to capture a significant difference. A change in FVC may be considered a harbinger of the changes in other lung volumes such as TLC. Also, data for spirometry parameters was available for a larger sample of our patients than those who had lung volumes and thus was more powered to reflect changes over the 2-year period.

Few previous studies have correlated lung function deficits and inflammatory markers in SCD [18, 35]. Similar to findings in our previous study of an inverse relationship between IP-10 and FVC [18], the present study showed changes in IP-10 to be negatively correlated with FVC changes for patients with ACS. IP-10 is the ligand of CXCR3 found on T lymphocytes (Th1), natural killer (NK) cells and eosinophils, and secreted by several cells such as monocytes, endothelial cells, adipose tissue, and fibroblasts [32, 36]. Our findings of associations of IP-10 with FVC, suggest a role of interferon gamma inducible chemokine responses in accordance with the murine study by Wallace et al. [37]. Patients in our ACS group showed elevated levels of IL-5, IL-13, and additionally, IL-6, a marker of monocytic inflammation, tended to negatively influence changes in FVC and FEV1 in patients with ACS. At least one mouse model investigating the effects of ova sensitization in SCD mice has demonstrated elevated inflammatory cytokines including IL-5, IL-13 and IL-6 [38], a similar inflammatory stimulus may be ongoing in our patients with ACS. Elevated monocytic inflammation in SCD has been established in several human and animal studies [38–41]. Elevated serum and sputum IL-6 has been reported in SCD patients at steady state and particularly with history of ACS [42, 43]. Due to relatively few patients with asthma and allergic rhinitis, the potential impact of allergic sensitization could not be investigated further in our study.

There have only been a handful of human investigations studying underlying inflammatory pathways and lung disease in patients with SCD [29]. Our study is novel as it attempts to connect lung function changes with inflammatory mediators, in children with SCD and ACS. Inflammation appears to be at the crux of SCD complications [30]. Identification of specific inflammatory pathways can inform and direct future interventional studies for management of SCD.

Asthma has been frequently reported as a comorbidity in SCD [24–28]. The diagnosis of asthma in SCD can be challenging given overlapping features of wheezing during ACS, use of bronchodilators in ACS, and misinterpretation of lower airway obstruction and airway hyper-responsiveness seen in SCD as asthma [25, 29, 44, 45]. A subset of our patients had asthma (28.6%), which is in the range of the estimated prevalence of 17–28% in SCD and within the range of asthma prevalence among African American children [29]. Asthma was recorded based on physician diagnosis. Patients with asthma had slightly lower FEV1 but no significant difference in other PFT parameters, including FeNO (results not reported). In our multivariate model, asthma predicted FEV1 and FVC. Our reported prevalence of asthma could have been an overestimate secondary to lower FEV1 and FVC.

In our multivariate model, age was negatively related to FEV1 and FEV1/FVC as can be expected with the natural course of decline in lung function. Males had lower FEV1/FVC and higher TLC which is likely secondary to gender based differences in the biological and mechanical properties of the lungs, and the concept of dysanapsis [46]. Higher lung volumes in males compared to females can impact flow rates, as these relate with airway size and lung volumes. Lower volumes characterized by less elastic recoil, drive equal pressure points further upstream, limiting airflow at higher flow rates and thus with higher FEV1/FVC ratios (seen in females).

The limitations of this study are its exploratory nature, small sample size and the potential effects of an intervention in the clinical trial that may have influenced cytokine levels and lung function. Vitamin D therapy has been shown to have a beneficial effect on reducing asthma exacerbations and respiratory infections [47, 48]. The postulated mechanism of action is through T lymphocyte mediated immune–modulation [49, 50]. Further, the literature supports evidence that cytokine levels are transitory with temporal variation. The number of patients with asthma and allergic rhinitis in this study was too small to derive meaningful conclusions. Another limitation of the study was the absence of T-lymphocyte quantification from peripheral blood mononuclear cells (PBMCs). A future prospective study can help to follow levels of cytokines over time along with PMBC analysis and relationship with changes in lung function. Quantification of airway biomarker levels in exhaled breath condensate can additionally help to delineate the inflammatory process in the airways. We further, did not have any data on vaping or tobacco exposure history for our patients to assess their impact on lung function, and this should be considered for future studies. Another limitation is the potential of generalizability of our results given that 50% of our patients were of Hispanic ethnicity. In the US, SCD occurs among about 1 out of every 365 Black or African-American births and among 1 out of every 16,300 Hispanic-American births, thus making Hispanic Americans the second largest ethnic/racial group with SCD in the US. Additionally, the majority of the population we serve are from Caribbean countries who often have African American ancestry. SCD is also very prevalent in many other Latin American countries. We also chose to not report on race, which is now accepted as a social construct rather than a biological construct.

Our present findings strengthen the association of ACS with lung function deficits and indicate that inflammatory mediators may be responsible. The current practice has been to associate most patients with SCD who have lung function defects and pulmonary involvement or ACS, with having asthma and managing them with standard asthma medications. A relationship of ACS with IFN induced cytokines such as IP10 and monocyte driven inflammation involving IL-6, is a step towards understanding airway inflammation in SCD patients following ACS, and its relationship with lung function changes. With more insight into the underlying mechanisms of ACS in SCD leading to pulmonary injury, future therapeutic targets can be directed toward more specific disease mechanisms.

## Supporting information

S1 Table S1A Table: Descriptive statistics of PFT parameters at baseline for two ACS group. S2B Table: Descriptive statistics of PFT parameters at 2 years for two ACS group.
(DOCX)

S2 Table Sensitivity analysis -multivariable GEE model for predictors of lung function parameters with SCD genotype and other covariates. GEE model with identity link and independence correlation structure to predict PFT parameters using age, sex, ACS, asthma, hydroxyurea, treatment arms and SCD genotype. Others for genotype include HbSC, HbSB Th + and HbS/HPFH.
(DOCX)

S1 Fig Change in cytokine levels from baseline to 2 years for ACS vs non-ACS groups. Boxplots for changes of TNFa, IP-10, IL-5, IL-6 and IL-13 from baseline to 2 years with p-value obtained from Wilcoxon rank sum test for comparison of changes of two groups.
(TIF)

S1 Data (CSV)
