# Supplementary material for: Acute chest syndrome, airway inflammation and lung function in sickle cell disease
Source: PLoS One. 2023 Mar 30;18(3):e0283349. doi: 10.1371/journal.pone.0283349 (PMC10062579; doi:10.1371/journal.pone.0283349)
Supplement: S1 Table — S1A Table: Descriptive statistics of PFT parameters at baseline for two ACS group. S2B Table: Descriptive statistics of PFT parameters at 2 years for two ACS group. (DOCX) [file pone.0283349.s001.docx]

| Parameters | n | Non-ACS n=15  Median [min, max] | ACS n=40  Median [min, max] | Wilcoxon rank sum test p-value |
| --- | --- | --- | --- | --- |
| FVC % predicted | 52 | 90.00 [58.00, 114.00] | 89.00 [57.00, 116.00] | 0.907 |
| FEV1 % predicted | 52 | 80.00 [57.00, 103.00] | 88.00 [57.00, 107.00] | 0.933 |
| FEV1/FVC | 52 | 88.00 [65.00, 96.00] | 85.00 [66.00, 98.00] | 0.849 |
| FEF25-75 % predicted | 47 | 70.00 [37.00, 160.00] | 68.50 [26.00, 176.00] | 0.910 |
| TLC % predicted | 37 | 95.00 [69.00, 134.00] | 77.50 [61.00, 114.00] | **0.034** |
| DL adj for Hb (ml/min^-1^mmHg^-1^) | 33 | 80.00 [51.00, 101.00] | 60.50 [45.00, 107.00] | 0.115 |
| KCO adj for Hb  (ml/min^-1^mmHg^-1^L^-1^) | 35 | 88.00 [71.00, 109.00] | 86.50 [57.00, 130.00] | 0.806 |

| Parameters | n | Non-ACS n=15  Median [min, max] | ACS n=40  Median [min, max] | Wilcoxon rank sum test p-value |
| --- | --- | --- | --- | --- |
| FVC % predicted | 54 | 90.00 [66.00, 129.00] | 90.00 [58.00, 111.00] | 0.938 |
| FEV1 % predicted | 54 | 85.00 [60.00, 110.00] | 83.00 [54.00, 111.00] | 0.657 |
| FEV1/FVC | 54 | 82.00 [69.00, 93.00] | 81.00 [64.00, 93.00] | 0.445 |
| FEF25-75 % predicted | 47 | 69.00 [50.00, 104.00] | 61.50 [12.00, 106.00] | 0.204 |
| TLC % predicted | 43 | 85.50 [69.00, 104.00] | 76.00 [61.00, 130.00] | **0.039** |
| DL adj for Hb (ml/min^-1^mmHg^-1^) | 42 | 73.50 [53.00, 101.00] | 67.50 [34.00, 96.00] | 0.690 |
| KCO adj for Hb  (ml/min^-1^mmHg^-1^L^-1^) | 42 | 85.00 [77.00, 112.00] | 93.00 [50.00, 136.00] | 0.248 |
