# Supplementary material for: Acute chest syndrome, airway inflammation and lung function in sickle cell disease
Source: PLoS One. 2023 Mar 30;18(3):e0283349. doi: 10.1371/journal.pone.0283349 (PMC10062579; doi:10.1371/journal.pone.0283349)
Supplement: S2 Table — GEE model with identity link and independence correlation structure to predict PFT parameters using age, sex, ACS, asthma, hydroxyurea, treatment arms and SCD genotype. Others for genotype include HbSC, HbSB Th + and HbS/HPFH. (DOCX) [file pone.0283349.s002.docx]

| Outcome | **FEV1** | | **FVC** | | **FEV1/FVC** | | **TLC** | |
| --- | --- | --- | --- | --- | --- | --- | --- | --- |
| Variable | Coefficient (95%CI) | p-value | Coefficient (95%CI) | p-value | Coefficient (95%CI) | p-value | Coefficient (95%CI) | p-value |
| Age at enrollment (years) | -0.837 (-1.69, 0.014) | **0.050** | -0.291(-1.12, 0.534) | 0.464 | -0.610(-1.05, -0.173) | **0.006** | 1.18(0.018, 2.35) | **0.047** |
| Sex  Male  Female | -0.077(-6.00, 5.84)  Reference | 0.980 | 1.11(-5.03,7.25)  Reference | 0.724 | -3.44(-6.60, -0.280)  Reference | **0.033** | 7.83 (0.456, 15.2)  Reference | **0.037** |
| Asthma  Y  N | -9.09(-16.5, -1.68)  Reference | **0.016** | -8.95(-16.4, -1.48)  Reference | **0.019** | -0.314(-3.95, 3.32)  Reference | 0.866 | -4.54(-13.1,4.03)  Reference | 0.299 |
| H/o ACS  Y  N | -0.203(-6.97, 6.57)  Reference | 0.953 | -0.035 (-7.89, 7.82)  Reference | 0.993 | -0.448(-4.53, 3.63)  Reference | 0.830 | -8.06(-18.3, 2.20)  Reference | 0.124 |
| Hydroxyurea  Y  N | 3.38(-5.16, 11.9)  Reference | 0.438 | 3.79(-5.97, 13.6)  Reference | 0.466 | -0.200 (-4.28, 3.88)  Reference | 0.923 | 5.36 (-3.28, 14.0)  Reference | 0.224 |
| Randomization  A  B | -6.30(-12.7, 0.071)  Reference | 0.053 | -7.56 (-14.3, -0.866)  Reference | **0.027** | 1.16(-1.99, 4.31)  Reference | 0.471 | -3.78(-11.8, 4.24)  Reference | 0.355 |
| Genotype  HbSS + HbSB Th0  Others | -9.30(-21.1, 2.55)  Reference | 0.124 | -8.48(-18.9, 1.95)  Reference | 0.111 | -0.239(-6.35, 5.87)  Reference | 0.939 | -15.1(-31.6,1.33)  Reference | 0.072 |
