# Supplementary figures and images for: Acute chest syndrome, airway inflammation and lung function in sickle cell disease
Source: PLoS One. 2023 Mar 30;18(3):e0283349. doi: 10.1371/journal.pone.0283349 (PMC10062579; doi:10.1371/journal.pone.0283349)

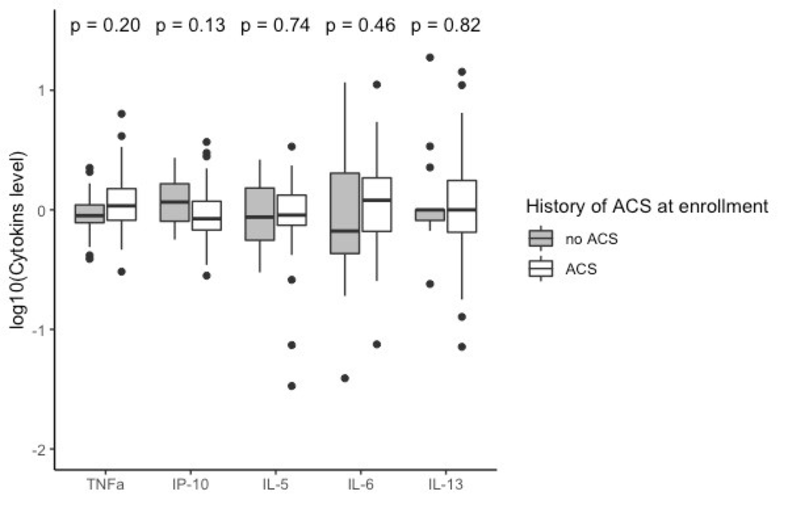

Supplement: S1 Fig — Boxplots for changes of TNFa, IP-10, IL-5, IL-6 and IL-13 from baseline to 2 years with p-value obtained from Wilcoxon rank sum test for comparison of changes of two groups. (TIF) [file pone.0283349.s003.tif]
